# Sea-Air and Sea-Bathing

**Published:** 1880-05

**Authors:** 


SEA-AIR AND SEA-BATHING. By John H. Packard, M D , Surgeon to the Episcopal Hospital, etc. Philadelphia: PRESLEY BLAKISTON, 1012 Walnut street. 1880.

